# Abandoning the SRO: Public Health Withdrawal from Sanitary Enforcement in Vancouver’s Downtown Eastside

**DOI:** 10.1177/00961442211018795

**Published:** 2021-06-04

**Authors:** Jeffrey Masuda, the Right to Remain Research Collective

**Affiliations:** 1 Queen’s University, Kingston, Ontario, Canada

**Keywords:** public health knowledge, policy failure, *cordon thérapeutique*, Vancouver, Canada

## Abstract

This paper situates a ten-year period of political upheaval in addressing the problem of Single Room Occupancy (SRO) housing in Vancouver, Canada, within an epistemic transformation of public health. Until 1970, the Vancouver Health Department exemplified a colonial history of public health in establishing the city’s skid road as a *cordon sanitaire*. But the 1970s saw a sudden fading of the Department’s authority just as a more collaborative approach to housing policy was emerging. The sunsetting of sanitary enforcement was driven in part by the arrival of a “new public health” that became primarily concerned with defining public health problems and solutions through the regulation of racialized bodies and behaviors—a *cordon thérapeutique*. By the 1980s, this shift constituted an epistemic and regulatory abandonment of SRO housing, leading to the accelerated deterioration of the entire housing stock and costing incalculable human suffering and the loss of lives.

## Introduction

On June 12, 2017, the City of Vancouver shut down the Balmoral Hotel, a run-down century-old Single Room Occupancy (SRO) building located in the heart of the predominantly low-income Downtown Eastside (DTES). The shutdown came as little surprise to most locals who have borne witness to its steady decay from decades of neglect. The Balmoral is owned by members of the Sahota family, the most notorious slumlords in the city, and the subject of a litany of media headlines going back decades.^1^ Eviction orders had come only ten days earlier, prompting a frenetic effort to re-house 173 low-income tenants in a context where both homelessness was at an all-time high and mortality from opioid overdoses was skyrocketing. Only one year later, on June 28, 2018, history repeated with the shutdown at the Sahota-owned Regent Hotel on the other side of Hastings Street facing the Balmoral, evicting a further 143 tenants.

Generations of tenants have suffered the precipitous decline in the state of repair of SRO buildings like the Balmoral and Regent, despite consistent efforts by community activists over the last half-century. While the Sahotas were subjected to much public vilification during both closures,^2^ their chronic negligence had been made possible only through consistent wavering in governmental SRO oversight, including a record of half-measures in both enforcement and reinvestment directed at stemming the erosion of their habitability going back decades. The city’s recent complicity ostensibly lay in part to the lack of enforcement of its regime of bylaws designed to uphold basic standards of health, safety, maintenance, and repair. In the months prior to the evictions, local housing organizer Wendy Pedersen had hoped one bylaw in particular would prevent the shutdowns altogether as they were designed to do, demanding that “the city should use a clause in its bylaws to undertake the necessary repairs, then bill the owners.”^3^ Pedersen was citing Section 23.8-11 of the city’s Standards of Maintenance Bylaw, which empowers the city to avoid condemning buildings by undertaking repairs in buildings that are in violation and billing the owners, with timely repayment assured through the threat of a tax sale.^4^ But it has been a point of endless consternation to local organizers that this bylaw provision has never been utilized since it was put into force on SROs in 1981.

There are nearly 4,500 tenants who live in the just over 100 privately owned SROs that remain today.^5^ Many of these SROs, like the Balmoral and Regent, have been on the precipice of closure for years and have been poorly managed, hazardous, and beset with violence and criminality. Yet, the problem of SRO housing habitability in the city has hardly registered in public health policy in recent memory, reflecting an absence of oversight that belies well-established accounts of its historic role in the sanitary regulation of housing that began over a century ago. Indeed, the Balmoral/Regent fiasco reflects a much longer “slow-motion” public health crisis in the DTES since the 1980s.

This paper examines the relationship between housing bylaw non-enforcement, the decline of SRO habitability, and the public health crisis in Vancouver through the prism of policy failure theory.^6^ Policy failure is read not simply as the ineffectiveness of a single bylaw, nor even to account for the widespread inability of twentieth-century public health measures to address the complex and contradictory challenges of the city.^7^ Rather the idea of failure implied here is that of a productive injustice—that is, the creation of the modern SRO slum—found in the epistemic *abandonment* of policies and practices prioritizing sanitary health first developed a century ago that suddenly disappeared after the 1970s. The bylaw can be read as a fulcrum in the production and interpretation of knowledge concerning the shifting role of public health in the regulation and racialization of urban places and human lives. Building on critical historical scholarship, such knowledge when put to work in places like the DTES is a form of colonial rule in which governmental technologies of sanitary surveillance and enforcement that dictated the creation of a *cordon sanitaire* in Vancouver were set aside under the increasing influence of neoliberal technologies of behavioral surveillance and control. Public health’s epistemic transformation of the DTES created a *cordon thérapeutique*, a novel concept that describes the abandonment of not only a bylaw but of a place through the medicalization of inner-city lives. The timing of such an abandonment, enacted through the selective relegation of policy, coincides in both time and political context with similar neoliberal governance shifts in the DTES that have undermined tenants’ rights and well-being for the past half-century.^8^

The Vancouver Health Department (VHD) played a shifting role in SRO housing leading up to its abandonment of habitability enforcement altogether at the turn of the 1980s. While previous critical scholarship has examined the earlier pre- and postwar period of the VHD’s establishment of the *cordon sanitaire*,^9^ more recent developments remain unexamined. Closer scrutiny of events of the 1970s will show how the shift to a *cordon thérapeutique* was (and is) an equally colonial apparatus, contributing to the establishment of what Nick Blomley describes as an outlaw zone.^10^ Recent critical interdisciplinary urban scholarship sees public health’s role in the city as a continuation of, rather than a disjuncture from, colonial urbanization over the twentieth century. Failure therefore occurred at a key historical moment that precipitated today’s SRO challenges; the nascent neoliberal public health knowledge mobilized to govern the DTES “at a distance”^11^ and ultimately leading to the VHD’s abandonment of SROs and the increasingly adverse health circumstances of tenants after 1981.

## Bylaw Enforcement and the *Cordon Sanitaire*, 1914-1969

The analysis of contemporary policy failure in SRO housing begins with studies on the co-constitutive history of municipal public health bureaucratization in Vancouver and processes of racialized containment in the DTES, then known as skid road,^12^ during the city’s early-to-mid-twentieth-century period of colonial urbanization. Critical scholarship has moved beyond institutional histories, to trace how public health discourses and practices have been shaped by and have given shape to urban form and function.^13^ Such historiographies see public health knowledge as an essential part of the colonial apparatus, providing a technology of spatial power premised upon principles of population surveillance, public hygiene, and a politics of social amelioration.^14^ For Alison Bashford, “Public health programmes and visions were a key way in which colonised people and territory were administered and came to be rendered intelligible to colonisers.”^15^ In the frontier city of Vancouver as elsewhere, such practices were critical in the colonial vision for an exclusively white settler society, yet built upon unceded Indigenous lands and heavily dependent on a workforce of predominantly Chinese and Japanese immigrants.

From the late nineteenth century, governing poor non-European immigrants and Indigenous peoples was synonymous with the sanitary agenda of early public health leaders, to be realized through the application of municipal bylaws directed at living conditions in racialized ghettoes in Vancouver. Bylaw inspection was implicated in the city’s earliest efforts to limit Asian immigration; Jill Wade documents the city’s sanitary inspector participating in the 1902 Royal Commission on Chinese and Japanese Immigration to demonstrate the putative incompatibility of Chinese living standards with the provisions of housing bylaws.^16^ The establishment of bylaws was instrumental to the exercise of colonial municipal authority; Dorothy Porter reflects on how the realization of the colonial city made the establishment of a public health bureaucracy possible through the epistemic authorization of medical knowledge that was both selective and exclusionary on the basis of class, race, and gender.^17^ By the interwar period, the maturing discipline of public health sciences influenced public health practices on the ground contributing to a white, masculinist urban vision by “naturally” cordoning off Chinatown and adjacent Japanese Canadian *Paueru Gai* through racist and pathologizing imaginaries of social inferiority, disease susceptibility, and xenophobia.^18^

As the city’s resource economy expanded, the built environment accommodated a rapidly growing and increasingly diverse transient labor force. Primarily, but not exclusively in the skid road area, single room accommodation was the norm, from basements and spare rooms in single-family houses, to cabins, to dedicated lodging houses and multistory hotels.^19^ Regulation of lodging houses allowed the early city to segregate a cheap labor force primarily of immigrants from Japan and China from European settlers. As Patricia Roy notes, “white British Columbians generally believed that Asians were protected by ‘our laws,’ but that they did, and *should*, live in ‘a world of their own’” [emphasis in original].^20^ According to Kay Anderson, the VHD played a major role in the creation and legitimation of racialized containment, writing and applying bylaws as a method of gradually delineating skid road into a colonial *cordon sanitaire* to assuage the fear that white Vancouverites held about the “degraded humanity from the Orient,”^21^ including their “loathsome diseases.”^22^

According to Margaret Andrews’ authoritative account, from the city’s founding in 1886, the ongoing challenges associated with the largely unplanned housing sector created a governable terrain that led to the establishment of the VHD, in part through the establishment of bylaws.^23^ Appointed in 1904, the city’s first full-time Medical Health Officer (MHO), Dr. Frederick T. Underhill aimed to solve the problem of housing habitability enforcement in the city’s expansive and arguably chaotic stock of lodging houses.^24^ Beginning in 1913, he undertook a model bylaw process that drew from notable British and North American cities, exemplifying a colonial impetus and technology. For Underhill, “What other countries have done, Canada can do, nay must do, if she is to retain her place in the foremost ranks of the peoples of the World . . .”^25^ While delayed by the war, Underhill was ultimately successful in creating the city’s first Lodging House Bylaw, passed by City Council in 1921. The bylaw provided a more secure status for the nascent Health Department relative to the more established Works Department.^26^ Underhill had insisted that the Lodging House Bylaw must be distinct from the Building Bylaw on the basis of the human-centered expertise that engineering lacked, pointing out that “A building may be of first-class construction but not fit for a dwelling.”^27^

To Underhill and his contemporaries, public health expertise began where bricks and mortar ended—where building bylaws were for *construction*, health bylaws were for *conditions*. Therein lay the latter’s role in racialized containment as they expressed as much as they enforced what constitutes an acceptable modality of habitation. The Lodging House Bylaw set a disciplinary frame of reference for constituting a “healthy” urban infrastructure premised upon minimum standards of habitability in accordance with British North American ideals. In mandating an orderly approach to housing design and maintenance dictated by sanitary considerations, the bylaw designated existing housing as inherently problematic and often on the basis of the assumed lifestyles of its residents. Advocating for a strong presence on the ground, Underhill insisted upon uniforms for health inspectors when on duty.^28^ In its rearguard application to the racialized enclaves of Chinatown and *Paueru Gai*, sanitary principles provided a means to designate places that were “unhealthy” through perceived disorder, the threat of disease outbreak, and general non-conformity to the domestic norms of the white population.^29^

The shift west in Vancouver’s downtown development over the first three decades of the twentieth century made matters worse as a gradual increase in the proportion of rooming house ownership and tenancy in the core area accommodated the concentrated growth of Japanese and Chinese Canadians. Also key to the changing environment, the management and maintenance of housing fell increasingly to these communities: by the end of the century’s third decade, nearly 50 percent of business licenses for lodging houses in the core area were held by Japanese Canadians, supporting large parts of the housing inhabited by Japanese Canadian workers and families, as well as laborers from diverse ethnic groups.^30^ This demographic and economic shift influenced the nature of interactions between public health inspectors and an increasingly racialized community of owners, managers, and tenants. The Lodging House Bylaw formalized the authority of the MHO and central role of health inspectors in intensive and effective, if intrusive, scheme of housing enforcement.^31^ Through the bylaw, health inspectors were mandated to provide routine surveillance over the tenants who occupied such forms of housing, leaving them more vulnerable to strict enforcement measures whose violation could end in eviction.

The expulsion of Japanese Canadians from the city in 1942 dramatically changed the social and built environment on skid road. But in many buildings, the good general upkeep of Japanese Canadian housing belied popular characterizations, ensuring that their legacy would live on in the built environment they left behind. This legacy was assured as early as July 1942, when city building inspector Andrew Haggart thwarted the efforts of Alderman George Buscombe, the notably anti-Asian chair of the city’s Special Committee on Japanese Property, to demolish the “Japanese District.” Haggart insisted that unoccupied buildings were “not structurally dangerous” and “no worse than some other sections of the City.”^32^ His intervention effectively blunted Buscombe’s efforts to guarantee the irreversibility of the uprooting, declaring that “the Department will not condemn buildings which are not structurally unsafe for private gain.”^33^

For Buscombe and others, the removal of the locally integrated Japanese Canadian community was motivated by a desire to reclaim at least one part of the *cordon sanitaire* for “white habitation”^34^; however, the uprooting had an unanticipated effect, ultimately creating the conditions for negligent management and requiring a significant intensification of bylaw enforcement against tenants and managers who replaced the Japanese Canadians. Although the consequences of ownership transfer remain poorly understood, the repositioning of a large part of the area’s lodging houses into new hands likely had the effect of reducing the level of residential attachment and internal cultural affiliation among those responsible for the maintenance and repair of much of the skid road housing stock after 1950.^35^ These changes contributed to decisions to further marginalize the area, including the closure of the streetcar line in 1955, and the end of service from the north shore ferry and Union Steamship boats in the late 1950s.^36^ Another significant contributor was the “aging in place” of—largely white—war veterans, pensioners, and other social groups left behind in the changing postwar economy but who found a refuge of affordability in the degraded housing environment. The demographic changes created a neighborhood that was no longer a haven for working-class resource workers. A later commentator described it as the “ashcan” of society.^37^

For the sanitary inspectors on the ground, this period represented a marked shift from a relatively straightforward, if ethically fraught, policy of regulating a largely integrated community and housing economy to one that mediated between external rent-seekers and an increasingly socially marginalized and exploited population. Bylaw enforcement remained a high priority in the postwar period, even as the VHD expanded into a fully fledged multidisciplinary bureaucracy following the rationalist and socially reformist ideologies of provincial leadership after 1933.^38^ Interestingly, while the new scientific aspects of public health medicine—audiometry, X-ray, and so on—became regionalized under a wider metropolitan authority, the more traditional practices of inspection and nursing remained locally funded and delivered.^39^ Now operating under a local health unit structure, detailed inspector records from Health Unit 1 beginning in 1943 reveal a consistent and intensifying surveillance campaign within the skid road area.^40^ Inspections in Chinatown describe interactions between landlords and tenants that show little room for negotiation. Enforcement resulted in fines and evictions under the health bylaws, and over time the shuttering and demolition of a substantial part of the housing stock in the DTES under the building bylaw.^41^

The rapid growth of the city after the 1950s, combined with an aging housing stock, kept sanitary inspectors busy, even while it seemed the problems were only worsening. Sanitary inspection priority lists from the late 1960s and early 1970s placed “Lodging House (licensed or not)—other than good quality” at the top of the list.^42^ Requiring a minimum of one inspection annually, lodging houses constituted nearly one-third of total premises subject to routine inspection across the city. With a cadre of about twenty staff, nearly 21,000 inspection actions were completed in 1967 alone, 4,389 were in “rooming houses.”^43^ By 1971, staff reductions resulted in an unsustainable population ratio of one inspector per 18,200 citywide, leading the VHD to undertake a work evaluation in 1972 as an effort to improve overall efficiencies and accommodate the increase in duties and focus on areas of highest need.^44^ But the lack of progress meant that something clearly needed to change.

## Adding Carrots to Sticks, 1970-1978

City documents confirm that by 1970, the economy of skid road had fully shifted toward meeting the needs of society’s “down and outs,”^45^ largely referring to single, unemployed white men who preferred the affordable accommodation and abundance of been parlors in the area. While such accounts tended to elide racial dynamics surrounding the area’s growing impoverishment, the worsening situation of the lodging houses—SROs in current parlance—was nonetheless an objective reality for both residents and those who surveilled them. This period of deterioration and isolation facilitated the arrival of the rapidly urbanizing population of Indigenous peoples, numbering nearly 20,000 in the city by 1976, 50 percent of whom were unemployed.^46^ But in failing to anticipate the housing impacts of this coercive urbanization of Indigenous peoples, long-standing problems of rural colonial exclusion were being translated into problems of urban colonial invisibilization. Rather, for the VHD, the problem of skid road became framed within a professional discourse that would imbricate its people neither in their susceptibility to sanitary risks nor their experiences of colonial trauma or racial and class-based segregation, but rather on the basis of substance use and corresponding behaviors. The arrival of a singular “chronic alcoholic” set the stage for a sea change in skid road housing action in the next decade. By 1967, a total of 3,239 single unemployed men on the welfare payroll were living in Vancouver, with the city’s MHO reporting a more than threefold increase in drinking-related offenses on skid road since 1950.^47^ By 1967, City Council had established a “Joint Committee re: ‘Skid Road,’” which met regularly to coordinate municipal departments’ (including VHD’s) respective efforts in the neighborhood.^48^ That same year, Council appointed a new MHO, Dr. Gerry Bonham, who would champion VHD’s housing file for the next decade.^49^

The summer of 1969 marked the beginning of a series of concerted efforts by the VHD to shift toward the medicalized perspective, even while inspection played an important rearguard role. A University of British Columbia (BC) medical student hired as a summer student in what was now the VHD’s North Unit conducted a study of skid road with an emphasis on the plight of the chronic alcoholic. C. A. Gill spent two months interviewing local residents, health workers, and housing operators, eventually generating a report that was consistent with a new public health mentality inclined to clinical intervention rather than inspectorial enforcement. Recognizing the “appalling” quality of housing, Gill endorsed an approach that would see housing used as a form of place-based therapeutic containment:

Since most indigents on Skid Road who require a place to stay have no serious intention of changing their pattern of living, compulsory rehabilitation-oriented services are futile. An alternative to present facilities would be to have a . . . building which would accommodate a large number of men, whose rent is looked after by the City Social Service Department.^50^

The medicalization of the DTES after 1970, corresponding to a sea change in public health discourse and practice, could be construed as the beginning of the end of public health’s historic mandate in the enforcement of a *cordon sanitaire* and its replacement with one inclined toward a *cordon thérapeutique* premised on governmental technologies of behavioral surveillance and control. Within a decade, this shift put an end to the health inspectors’ primary role in housing bylaw enforcement in Vancouver.

Gill’s report prompted the next invocation of VHD authority on the concentrated problem of skid road that initially retained the know-how of the health inspectors, but gradually led to their marginalization. Housed in the Department of Social Planning/Community Development, a new Local Area Coordinator’s top priority was to “survey the present available accommodation, the skid road population, behaviour problems and transiency”^51^ that relied heavily on VHD inspectors and MHO Bonham’s sometimes conflictive leadership.

The sequence of events in taking up the housing problem after 1970 was swift and turbulent. The new approach acknowledged a need to match bylaw enforcement with housing reinvestment—a carrot to match sticks. A May 1970 report of the Vancouver Board of Administration placed the “purchase and conversion of existing housing” last on a list of priorities to secure federal housing support.^52^ This was the first instance of the city’s decade-long pursuit of the carrot and stick approach to SRO rehabilitation in the DTES that, at least at first, relied on the eyes and ears of VHD inspectors working on the ground.^53^

A July 1970 meeting of representatives of the health, police, and social planning/community development departments charged inspectors in the VHD North Unit with implementing a two-part survey of demographic and built environment characteristics of the area.^54^ The resulting report, *Downtown Eastside*, published in May 1971, characterized the demographic profile of the DTES and the number, types, size, and condition of dwelling units, painting a grim picture of the state of housing: 33 percent of nearly 7,000 residential buildings included in the survey had received health bylaw violations and 24 percent of interviewees had “serious complaints” about their housing conditions.^55^ A new phenomenon was the finding that rising rents were being driven by an increasing rate of building closures, leading to a high level of internal displacement among long-term residents.^56^ Clearly, a new approach was needed.

Alarmed by the findings, City Council requested two follow-up reports, first on “the problems of persons forced to relocate because of the demolition of their dwelling units” and second on the “means of improving housing conditions in the Downtown Eastside.”^57^ Both requests led to the publication of an amalgamated *Skid Road Report*, delivered on November 18, 1971. Portending the future decentering of the VHD’s traditional role in housing enforcement, a reporter described it as a “unique session,” where “nine civic department directors sat around the conference table with the aldermen for an exchange of ideas.”^58^ Reflecting the same finding as the Board of Administration from a year earlier, the report concluded that a strategy of strengthened bylaw enforcement must be tied to measures to protect and rehabilitate the housing infrastructure.

The report called for an increase in fines for violations of the Lodging House bylaw and the creation of a competency-based landlord licensing system that would be issued by the MHO. Not mentioned in the report but apparently discussed at committee was a new housing standards bylaw, prompted by changes to Provincial legislation, which had been designed for citywide application. A reporter covering the meeting noted that the bylaw was proposed to work alongside a “method to have grants or loans provided to building owners to upgrade facilities,”^59^ although there was little appetite for such a program among Aldermen at the time.^60^

In the absence of an identifiable funding mechanism to incentivize structural rehabilitation, the total effect of municipal efforts in the early 1970s was to increase pressure on health inspectors to address worsening housing disrepair within their existing capacity. This go-alone approach in the context of a newfound aversion to condemning buildings and a rapidly changing political context proved counterproductive. A February 10, 1972, article in *The Province* reported on a VHD “crackdown,” citing twelve charges against operators and fifteen shuttered “flophouses” under the Lodging House Bylaw, with an unprecedented fine of CAN $300 given to one landlord.^61^ Internal VHD documents reported a total of 400 units lost.^62^ It had become clear that an enforcement policy on its own was not going to work, and thereafter the role of the VHD became increasingly tenuous.

The replacement of the long-governing and conservative-leaning Non-Partisan Association (NPA) party by the more centrist The Electors’ Action Movement (TEAM) party in the civic election of December 1972 hastened the changes to come. Within weeks of the election, TEAM Councilor Michael Harcourt, who had earlier become chair of the Interdepartmental Committee on Skid Road Housing, began courting the federal Canada Mortgage and Housing Corporation (CMHC). He was anticipating the passing of the federal National Housing Act by the Trudeau government, “with all the goodies it has promised for rehabilitation.”^63^

Harry Rankin, another newly elected Councilor from the leftist Coalition of Progressive Electors (COPE) party, was working with the director of the Welfare and Rehabilitation Department, W. Boyd, on a stepwise plan to identify and provide subsidies to SROs with reputations for better conditions and management.^64^ Rankin recognized that considerable costs to purchase and renovate properties would require a horizon of ten to twenty years to be realized, and therefore interim actions were needed. It was also widely recognized that many residents in the area were not amenable to any plan that would have them move into public housing, as they preferred to remain in the places that they lived—a recurring theme noted in earlier reports.^65^ Throughout this formative stage, North Unit health inspectors including one David Morgan remained at the table due to their expertise on SRO conditions and management relations, albeit now in a more subordinate role.

During the same period, the DTES community was finding its organizing feet by directing attention to public health oversight. Modest initial efforts began with a city-sponsored and federally funded program called the People’s Aide Association, housed at First United Church in the heart of the DTES. Created in 1972 by former First United community worker Peter Davies, by then working for the city’s Social Planning/Community Development Department, People’s Aide hired longtime area resident and steelworker Bruce Eriksen, who quickly radicalized the program’s approach before going on to become the “big man” of community organizing in the neighborhood and later a COPE City Councilor.^66^ In response to the acceleration of city actions on skid road, Eriksen held the VHD’s feet to the fire to ensure that “lofty sentiment” would be translated into meaningful change for residents, lest, as he and fellow People’s Aide worker Calvin Sandborn put in a February 19, 1973, open letter to the Mayor, they be forced to “burn the City Health By-Laws to warm themselves in freezing rooming houses.”^67^

Sandborn and Eriksen were adamant that adding more health inspectors would be futile unless they be empowered to enforce continuing $50 fines against slumlords, a bylaw measure that he claimed had “been lying unused on the lawbooks for 15 years.”^68^ In a critique of the VHD’s crackdown, they also appealed for a policy to “clean up, not to shut down” the 150 SROs that remained open.^69^ Initially, they sought a commitment to strengthen and enforce the city’s bylaws, filing complaint reports to the City Prosecutor on eight SROs they deemed in violation of sections of the Lodging House Bylaw.^70^ The chief Constable forwarded their concern to MHO Bonham, with the likely effect of further adding to the pressure on the inspectors.^71^

It appears that the community’s efforts had an effect. Council ordered Bonham on April 10, 1973, to redraft the Lodging House Bylaw with strengthened provisions.^72^ Reflecting the more collaborative civic environment, the MHO sought input from several DTES organizations, including the fledgling Community Legal Assistance Society.^73^ Most notable in the proposed new bylaw was the requirement for landlords to obtain competency-based operating permits to be issued by the MHO.^74^ It may be the success of the early campaign that played a role in Eriksen’s further politicization. By August 1973, Eriksen had convinced Davies to call a public meeting at First United, wherein the Downtown Eastside Residents’ Association (DERA) was formed.^75^ The moniker of skid road was hereinafter anathema; the neighborhood was now to be referred to only as the DTES.^76^ For the next several years, DERA became the most prominent community voice on the SRO housing file. In pushing for investment carrots to go with bylaw sticks, the relationship between DERA and the VHD became one of agonistic cooperation.

Prior to passing the bylaw amendment, the Council directed MHO Bonham on October 23, 1973, to “report on any additional staff required or ramifications necessary to enforce the (revised) Lodging House Bylaw.”^77^ In his report, Bonham requested staffing increases to support the operating permit program, as well as for joint inspections with the Fire Department. Council passed the revised bylaw on November 6, 1973, setting in motion a “program of inspection with emphasis on maintaining public health,” which was subsequently referred to as the “Lodging House program.”^78^

Exemplifying the old approach, Bonham pushed for intensified surveillance and enforcement of buildings throughout the mid-1970s, consistently advocating for a larger health inspection workforce and stronger bylaws. But within three months of the onset of the Lodging House program, the approach was yielding disconcerting results. A March 29, 1974, progress report to the Board of Administration outlined the intensity of the unit’s inspections within the first few months since the bylaw was passed: 120 initial letters of violations, 1,500 inspection reports, 61 short-term orders to correct structural problems, 4 cases forwarded to prosecution, and, as at the time of the report, a further 303 units lost in the year since the previous crackdown.^79^

These impacts were concerning to DERA, who increasingly saw the futility of the VHD’s enforcement approach. As reported in the November 1975 issue of their newspaper, *The Downtown East*, they castigated the city for its reluctance to impose stronger fines and to carry enforcement through to prosecution.^80^ Morgan, who was seen to be something of a mediator between DERA and the city,^81^ did not disagree. Quoted in the same issue, he said it is “more profitable for an operator to not maintain his premises and pay a small fine every year.”^82^ Likewise, in another piece in the same article, Alderman Rankin wrote that “To spend $50 or $100 for a fine rather than to spend $500 or $1000 to clean up or repair premises is good business.”^83^ At the very least, this sometimes-tenuous alignment of community, bureaucratic, and political voices appeared to portend changes to come in establishing the case for a stick and carrot approach as well as the declining role of the VHD within it.

Bonham’s response to DERA’s pressure with a new level of urgency to the situation of SRO safety and habitability was only matched by his own sense of futility of a go-it-alone approach, including what he saw as halfhearted support from Council. A December 10, 1975, update to Council’s Standing Committee on Housing and Environment summarized his frustration over the lack of progress from the previous three years of enforcement efforts. In the report, Bonham laments that with additional inspector hires granted the previous year, amid ongoing staffing challenges, “an increased lodging house program was [only] *temporarily* feasible” (emphasis added).^84^ The MHO’s report went on to state that the VHD

is not anxious to build any empire directed to the Lodging House program. The staff who have done a difficult job so well do not feel that they are fully utilizing the full range of their 2½ year training and find this work frequently exasperating.^85^

But as will be seen, the push to move the VHD out of its historical role in enforcement was to be accompanied by a pull toward a new mentality of intervention that led to the sidelining of health inspection altogether.

## Withdrawing from SRO Health Inspection, 1978-1989

The carrot and stick idea gained traction within the context of increasing community pressure, media attention, and more formal interdepartmental and intergovernmental collaboration. But the role of the VHD faded from prominence at the moment that a stronger stick, and perhaps more importantly public health leadership in housing, was needed most. What ensued after 1978 was a concerted effort to link Lodging House bylaw enforcement to efforts by DERA and civic officials to bring federal dollars to the table through the federal Rental-Residential Rehabilitation Assistance Program (Rental-RRAP). Established in 1973, RRAP was part of a national strategy to preserve Canada’s aging housing infrastructure, but a technicality in the program had left out privately owned SROs.^86^ It took several years and numerous advocacy efforts on the part of politicians and DERA alike for this oversight to be rectified.^87^

At first, Bonham and the VHD found a basis for cooperation with DERA, who had earlier embarked on their own campaign to bring stronger bylaw enforcement to the lodging houses. On June 9, 1977, DERA advocated to Alderman Rankin, their natural ally on Council, that more health inspectors be hired and authorized to use the city’s new Standards of Maintenance Bylaw to impel landlords to “rectify illegal conditions” (i.e., today’s Section 23, then Section 6 of the pre-amalgamated bylaw), citing once again the protracted and ineffective prosecutorial strategy that was required by the Lodging House bylaw.^88^ But Standards of Maintenance enforcement fell to property use inspectors in the Department of Permits and Licenses. Critically, its modus operandi was complaint based rather than the intensive routine approach of health inspectors. These differences quickly proved fatal for the VHD’s role in SRO enforcement.

Concerned that the VHD was grossly understaffed and underpowered, DERA began something of an insurgent inspections process, surveying forty rooming houses to point out the urgency of the problem of deteriorating conditions in hopes of impelling action.^89^ Also that summer, DERA collected 550 signatures for a petition to demand more health inspectors for the DTES.^90^ Prompted by DERA’s actions, a June 23, 1977, report to Council from the Standing Committee on Community Services references Bonham’s report from two years earlier in an effort to convince Council to hire additional inspectors.^91^ Once again, Morgan is quoted, this time in the *Downtown East*, “You’ve got to be on their (the landlords’) backs.”^92^ One month later, Council voted in favor of the proposal to “return to the lodging house inspector standards of 1974–75.”^93^

Unfortunately, the climate of tenuous cooperation between the VHD and DERA did not last long. In early March 1978, DERA submitted a report demanding action against landlords, based on complaints they compiled in twenty-five premises. Viewing their campaign as a “stab in the back,” Bonham had inspectors revisit all twenty-five premises, finding their concerns to be “not valid” and “overstated.”^94^ He also positioned the VHD in opposition to Council, which he accused along with DERA of hampering the efforts of inspections staff and of “dithering” on the SRO housing file altogether.^95^ Despite a sense of resignation, Bonham kept up his campaign, reporting in May 24, 1978, on the closure of two SROs totaling seventy-eight rooms due to repeated offenses under the Lodging House Bylaw.^96^

By the late fall, Council appeared to be ready to take bold multipartisan action on a new approach to a new housing program that had been developed by the Standing Committees on Community Services and Planning and Development. Critical to the public legitimacy of the program was the extent to which bureaucrats consulted with several delegations, including both DERA (representing tenants’ interests) and the BC Hotel’s Association (representing landlords’ interests), both of whom endorsed the plan.^97^ On November 7, 1978, Council approved the Committee’s recommendations, formally establishing the Downtown Housing Implementation Committee (DHIC), comprised of representatives from Health, Fire, Permits and Licenses, Planning, and Social Planning departments.^98^ Its initial mandate was to “develop and expedite a comprehensive and coordinated plan,” subsequently referred to as the Downtown Housing Program (DHP), focused specifically on matching enforcement to reinvestment.^99^ Charged with improving enforcement of bylaws, the DHIC was also instructed to bring both provincial and federal governments to the table with commitments to improved social assistance levels (under provincial jurisdiction) and to work directly with hotel and rooming house owners and operators to “develop a modified (federal) Residential Rehabilitation Assistance Program.”^100^

But on the eve of the birth of the DHP, the VHD’s leadership suddenly faded from view. While internal VHD records indicate both Bonham and Morgan were present during the November Council meeting, it appears from the minutes that they did not contribute to the deliberations.^101^ Two months later and likely reflecting his exasperation from earlier in the year, Bonham was no longer working for the City. After eleven years as MHO, during which time the DTES housing file had been a signature focus, he had taken a new position with the provincial government, leaving Morgan alone to represent the VHD on the DHIC.^102^

The sudden void in MHO leadership on the DHIC may have played a role in the ensuing withdrawal of frontline health inspectors from their historical role in routine housing bylaw enforcement. DHIC’s first quarterly report, delivered to the Standing Committee to Council in a split session on March 22 and April 3, 1979, provides a detailed synopsis of these events. The DHIC Chair Doug Purdy, deputy director of Social Planning/Community Development, was attempting to establish an argument for a “unidisciplinary” approach to inspections, citing the previous shortcomings in Lodging House Bylaw enforcement efforts noted above.^103^ In response to concerns raised at Council at the March 22 meeting, Purdy elaborated in a letter to the Committee his case for a new enforcement approach under what he called the “Housing Standards for Older, Multiple Residential Buildings By-law.”^104^

His core argument drove a stake into the heart of the VHD’s long legacy of enforcing the *cordon sanitaire*:

The existing Lodging House by-law which has been the primary tool applied by the City to ensure a sanitary standard in residential hotels and rooming houses contains a major deficiency—it does not contain the power to invoke the application of other appropriate by-laws for upgrading purposes.^105^

He was referring to the provision for mandatory repairs billed to landlords referred to in the introduction of this paper, and the ultimate realization of his proposal was a revised Standards of Maintenance bylaw. But rather than have VHD health inspectors take a lead role in a strengthened and ostensibly more coordinated unidisciplinary approach, he concluded,

it is felt that a consolidated enforcement approach will be improved by transferring some Public Health Inspectors to the Department of Permits and Licenses. The involvement of so many different By-laws is simplified by the adoption of this By-law and the Inspection Task centralized in one Department.^106^

The details of his plan revealed that this transfer was to be a temporary secondment, long enough, it was argued, for health inspectors to pass along their knowledge and skills to the property use inspectors on the health dimensions of lodging house surveillance and enforcement.^107^

The rationale for why Permits and Licenses, whose experience in Standards of Maintenance enforcement was at the time less than five years, none of which related to SROs or to issues of livability, should replace a more than half-century of VHD inspections can only be speculated upon. But its effect on the leadership and arguably morale among the VHD’s health inspectors is clearer in the historical record. The minutes to the April 3 session of the Council meeting suggest a rearguard effort by Morgan and Bonham’s replacement, acting MHO Tim Kinloch:

The Acting MHO suggested that integration of by-law enforcement staff will not result necessarily in better enforcement; that rewriting of current by-laws must occur first. He also suggested that if Health inspector positions are transferred away from his department, the department may not have sufficient manpower to carry out its routine health inspections with the same frequency as now . . . The Director of Environmental Health [Morgan] added that the problem of poor housing is not just a matter of poor building conditions, that problems of “hard to house” tenants and bad management also must be examined.^108^

It is revealing how Kinloch and Morgan’s appeal attempted to link both old and new public health mentalities of sanitary enforcement and behavioral management. But interdepartmental animosity may have lent support to Purdy’s effort toward consolidation. The Deputy City Manager concluded,

on the matter of Health inspectors being seconded to the Permits and Licenses Department, a lot of time was spent on this question; that the original proposal to do this was put forward by former MHO, Dr. Bonham, some two years ago.^109^

This statement misconstrued Bonham’s earlier position, as he had been pleading for more, not fewer, inspectors.^110^ Ultimately, the Committee recommended the transfer, which Council approved, with provisions for a progress report within a year.^111^

The funding strategy of the DHP came as an advocacy-based campaign for provincial subsidies and modifications to the CMHC Rental-RRAP. Changes to bylaw enforcement became the major focus of efforts by staff, who opted for consolidation. Given the staffing secondments to Permits and Licenses, the simplest approach was to cut and paste the health provisions of the historic Lodging House bylaw into the new Standards of Maintenance bylaw and to repeal the former in 1981. The consolidated bylaw would be utilized on a proactive basis only in the DTES area as the stick in a Comprehensive Inspection program tied to the DHP. This DHP therefore represented a fundamentally new regime of bylaw enforcement, departing from a concern for the maintenance of sanitary *hygiene* to one of triaging and valuating *property*.

The transition of duties and concomitant evacuation of health inspectors from housing inspection did not go uncontested. An April 1980 letter from Tim Roark, BC Chair of the Canadian Public Health Inspectors Association, proved to be prescient:

A move for public health inspectors from the Health Department to the Permits and License Department would severely impede the effectiveness of the “public health team.” Not only does the public health inspector enforce the provisions of the Lodging House Bylaw, but as a member of the Public Health team he [*sic*] has always been concerned with the health of the people residing within these premises.^112^

The Association recognized that the inspector was not simply a technical aid but in their role in bylaw enforcement also carried the powerful and historically wielded authority of the MHO and the collaborative approach to health protection. Kinloch refused to sign a DHIC progress report out of a concern that property use inspectors were not receiving adequate health-related training.^113^ Regardless of these concerns, by the time of their third report to Council on June 23, 1980, the DHIC had commenced with aligning the bylaws to their new staffing arrangements. In the meantime, the inspectors continued to be exasperated by their new roles within Permits and Licenses, reporting,

Over the past two years, the regular Lodging House Enforcement Program has been able to maintain but not significantly improve the standards of livability of accommodation in the core area. This reinforces the need for a combined program of City Bylaw Enforcement and RRAP financial aid to landlords.^114^

Possibly reflecting VHD’s obstructionist views on the effort to consolidate inspections, Morgan’s role in the DHP was reduced to working on a lodging house operators’ certification program intended to mitigate landlords’ negligence toward their bylaw responsibilities. Rubbing salt in the wound, he was instructed to inform the 911 operator that public health inspectors’ after-hours emergency telephone numbers were to be replaced by those of property inspectors working in Permits and Licenses.^115^ Finally, on December 16, the new Standards of Maintenance bylaw went to Council, just in time for that month’s civic election.

The election of December 1980 not only represented the sunsetting of the TEAM era but, particularly significant, brought DERA President Bruce Eriksen onto Council. By 1981, the Downtown Housing Plan, rebranded as the Downtown Eastside Housing Upgrading Program (DHUP), was the realization of the long-sought stick and carrot approach designed to secure the sustainability of the DTES SRO housing stock.^116^ The stick was now the Comprehensive Inspection Program, enforced by the strengthened Standards of Maintenance Bylaw, used as a means to identify priority buildings and incentivize landlords into the program through the issuing of repair orders exercised through Section 23. Over the next two years, the program encompassed a total of sixty-five lodging houses, totaling 1,974 units, undertaken by property use inspectors.^117^ VHD health inspectors, on the contrary, had been reduced to occasional consultants.

Rental-RRAP was the long-anticipated carrot, which by then had been improved and made applicable for the entire DTES area. The essential role of Rental-RRAP was its ability to tie federally subsidized renovations through forgivable loans and grants to fifteen-year vacancy controls designed to preserve the affordability of the housing stock. Over the next several years, the funding available through the Rental-RRAP program grew to the point that by the end of 1989, thirty SRO buildings in the DTES (roughly 20% of the building stock at the time) had undertaken major structural repairs, securing improved conditions and affordable rents in over 1,500 units. Critically, city reports concluded that the Rental-RRAP program had a significant impact on improving housing habitability for low-income people in the city.^118^ What is more, the key outcome of the program was expressed in terms of not only the structural dimensions of buildings undergoing improvement but, in the context of the DTES specifically, the greatly improved “livability and safety” of participating SROs. Yet, such assessments were made without dedicated public health presence on the ground.^119^

While the DHUP program’s success was objectively laudable, its failure lay in its short life span. On April 27, 1989, Rental-RRAP was suddenly canceled in the Conservative government’s federal budget.^120^ Both municipal and provincial politicians undertook a flurry of multipartisan efforts over several months to reverse the cancellation.^121^ Contra justifications offered by Allan Redway, the federal Minister of State for Housing, claiming that the program had failed to deliver for those in core housing need, the city offered statistics confirming that 698 units of affordable rental stock had been structurally upgraded in the previous three years alone, comprising 70 percent of the city’s Rental-RRAP funding.^122^ Citing the collaborative approach, the city manager report stated, “The Rental RRAP assisted the enforcement and upgrading program for the Downtown Eastside rooming houses and has been an important asset in raising housing standards for low-income tenants.”^123^ The Mayor took these arguments to Ottawa himself, requesting meetings with both Minister Redway and Prime Minister Brian Mulroney.^124^ The advocacy efforts were to no avail—Redway’s response to the Mayor revealed the neoliberal motivation behind the termination of the Rental-RRAP: “Efforts to reduce the federal deficit have meant that many difficult choices have had to be made. The termination of the Rental RRAP on April 27, 1989 was one of these.”^125^ Reading between the lines of the 1989 budget, the termination of Rental-RRAP was an early indication of the broader shift toward the neoliberal ownership society that emerged over the next decade across the North American continent.

## Epistemic Abandonment and the New Colonial Governmentality

The termination of Rental-RRAP was a sudden end to a long-standing “potential history” of public health intervention on SRO housing.^126^ Rather than culminating in a shift toward more sustainable housing reinvestment, the demise of DHUP resulted in the further erosion of SROs and a worsening health crisis in the DTES since 1990. But the preceding analysis shows that this policy failure not only implicates the funding cut itself but also to the abandonment of VHD’s commitment to SRO sanitary enforcement more than a decade earlier. Moreover, the rearguard effort of political leaders to reverse the funding cuts held little sway in the absence of an authoritative public health voice that might have argued for the program’s health benefits. The balance of this paper addresses not just what took place but how the abandonment of the VHD over SRO housing was facilitated by the epistemic shift toward behavioralism that ultimately pulled the VHD into a new colonial apparatus for the regulation of urban space and bodies and that subsequently disregarded housing altogether.

As stated earlier, the analysis of this shift moves well beyond descriptive institutional histories of municipal politics and toward an exposure of how epistemic influences from above influenced and were deployed by individual actors on the ground. This analysis draws specifically from Alison Bashford, for whom the arrival of the “new” public health after the mid-1970s represents a genealogical transition from “old” colonial public health practices of racial containment, toward new colonial governmentalities predicated on the “mapping of racial segregation onto health segregation.”^127^ From this perspective, the abandonment of housing bylaw enforcement after 1981 is an exemplary illustration of this genealogy as it saw VHD authorities pulled away from their historical responsibilities just as neoliberal influences ensured the eventual demise of the promising carrot and stick approach of the DHUP. The evidence suggests that these were convergent moments.

During the mid-1970s, turbulent epistemic undercurrents of public health appear to have had profound implications for the continued leadership of the VHD on the housing file. With the local housing crisis quickly accelerating and the VHD responding along largely traditional lines, another much more broadly scaled “epistemic crisis” was brewing within federal and provincial political epistemic communities that resulted in sudden and major shifts in public health institutions and practices across the globe. The historical and political context of the “healthcare crisis” prompted by Canada’s Medical Care Act of 1966 is amply documented elsewhere,^128^ so will only be summarized briefly here.

Responding to the growing chorus of alarmist voices about the simultaneous escalation of health care costs and diminishing returns on investment, in 1974, Federal Minister of Health and Welfare Marc Lalonde instigated, via the release of the think tank report, *A New Perspective on the Health of Canadians*, a radical transformation of the fundamental premise of public health.^129^ Distinguishing itself from an ostensible “old” public health centered on sanitary surveillance and enforcement, events of the 1970s saw to the emergence of a “new public health” premised upon a more persuasive approach within governmental practice that was highly influenced by advances in epidemiological and behavioral sciences.^130^

Starting in the second half of the 1970s, public health institutions embraced these twin disciplines to take their place within a biomedical revolution that had captured imaginations across the wider health care sector.^131^ The new approach valorized coupling population-based measurements of human illness with individualized lifestyle-based programs designed to be mobilized en masse to change population health outcomes. This approach represented a gravitational epistemic shift not only in how public health was re-conceived to prevent disease and promote health through persuasion. This epistemic shift quickly came to restructure radically public health institutions and practices on the ground in places like Vancouver.

It is not a coincidence that the gradual disappearance of VHD from the DHP after 1978 occurred just as the Lalonde report had begun to metastasize within the local public health bureaucracy, casting work on sanitary enforcement to the periphery in favor of the new approaches.^132^ From the inside, VHD reports reveal how the city was to be a model for the delivery of a Lalonde-style “Lifestyle Program”:

There is an exciting opportunity to deal with extremely important health problems. The major causes of death are due to unhealthy lifestyle. The Department, within existing resources, has explored the opportunities for developing a system for successful lifestyle change programs. To take advantage of new opportunities in the area of preventative health care more staff resources are needed . . .The Department of National Health & Welfare appears willing to fund a partnership within the City of Vancouver in Developing a model program. Federal participation is planned to commence January 1, 1978.^133^

The new methods were reflected in early public facing documents. A 1976 VHD document, *You, Vancouver: What your city health department knows and does*, presaged a revolutionary new ethos for the department that very much reflected the zeitgeist of the Lalonde report in applying the new behavioral science toward problems of fiscal unsustainability:

Sometimes just an attitude can make a big difference. At your Public Health Unit we’ve had a quiet revolution in the past couple of years. In our “bureaucratese” it sounds like, “try for a client-centred, rather than agency-centred approach.” It means we’re trying to build you up, to help you with your problems, rather than promote, or sustain, or build ourselves up . . . You know, and we know, that your most valuable possession, and our country’s greatest resource, is yourself.^134^

**Figure 1.**
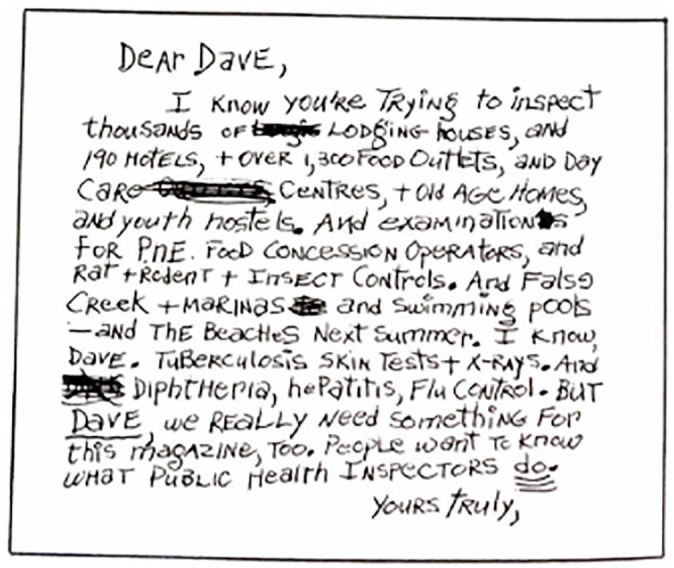
Anonymous mock letter to “Dave,” public health inspector. *You, Vancouver: What your city health department knows and does* (1976).^135^

From a historical perspective, the influence of *A New Perspective* on the VHD corresponded in time and practice to a shift from its long-standing role in upholding a colonial *cordon sanitaire* via bylaw enforcement to one that would establish a *cordon thérapeutique* premised upon the medicalization of both the DTES as a place and of its people through behaviors deemed as deviant from a white, middle class perspective. Over time, the public health revolution at a national level contributed to a very different understanding of health priorities that resulted in new responsibilities for the VHD in the DTES based on medical risk factors and behavioral persuasion and control. The place of housing and the role of inspectors are presented in the 1976 document only in parody: a back-page mock letter describes the predicament of inspector Dave Morgan cited earlier, too encumbered in his old responsibilities to contribute to the new priorities (Figure 1).

The behavioral approach suited the times: in light of the economic recession that had taken hold in the city, acting MHO Kinloch acceded to the reality that a focus on lifestyles represented the most important and practical response to the present climate of austerity that had arrived at all levels of government. As conveyed in his introductory remarks to the VHD’s 1980 annual report:

The present and foreseeable economic situation will limit the resources available such that only work of the highest priority ought to be done, and good information is necessary for good priority setting . . . We know that the major underlying determinants of preventable early death in Vancouver are cigarettes, excess alcohol, stress, accidents, poverty and poor nutrition.^135^

Notably, neither the DHIC nor the role of seconded public health inspectors in the DHUP is mentioned by Kinloch, nor in any subsequent executive and public health inspection staff meetings thereafter. Kinloch was seen to be a major proponent of “saving money by promoting healthy lifestyles”^136^ that in the DTES pointed squarely to the problem of chronic alcoholism.^137^ Thereafter, the role of health inspectors in the VHD was limited to less politicized environments such as swimming pools and restaurants.

The new climate in the VHD was lamentable. By December 1982, Kinloch had been fired, following an ongoing dispute with the City Manager related to the controversial fiscal restraints being foisted on the VHD by the new Council.^138^ VHD records during this period reveal a department in turmoil, plagued by low morale and without a clear focus. A July 1983 memo indicates staff in the North Unit advocating for a stress management workshop, focusing on a “problem solving process, intended as a helping thing, [as a] result of changes in the Vancouver Health Department and the toll taken on the staff.”^139^ Within the year, Kinloch’s replacement, another VHD clinician, took over the acting MHO role. Ted McLean, formerly Clinical Director, continued to oppose the City Manager’s austerity agenda, who at the time was endeavoring to strip the MHO position of its administrative authority.^140^ The shift was sudden and clear: By 1984, the AIDS crisis had landed in Vancouver and a permanent MHO was hired who had no recollection of VHD’s role in the development of the DHUP through the 1980s.^141^

## Re-situating Bylaw Non-enforcement

The fate of public health inspection in Vancouver’s SROs after 1981 provides at least a partial explanation to Wendy Pedersen’s predicament in 2017: the perplexing non-enforcement of Section 23 of the Standards of Maintenance Bylaw. Adapted in 1980 specifically for DTES SRO housing, the bylaw was rendered effectively obsolete after the demise of DHUP in 1989. Ironically, the Balmoral was one of four SROs identified as “worth rehabilitating” at the outset of DHUP, although it is unknown at the time of this writing whether the Sahotas received Rental-RRAP funding.^142^ Originally designed to serve as the “stick” for a significant program of SRO rehabilitation tied to federal “carrots,” Section 23 was never envisioned by its designers as a stand-alone enforcement mechanism.

But the more important history is of epistemic abandonment; the shift from colonial enforcement to colonial persuasion through the implicit disavowal of established standards of sanitation inspections in favor of a morally tinged focus on behavioral control seen to be typical of the DTES population. The impacts of Canada’s embrace of neoliberalism in housing policy and on public health are both well established but distinct areas of scholarship. The present study ties these two threads of neoliberal history directly together. Vancouver’s SRO history exemplifies Canada’s failure to ensure a basic standard of housing since the onset of neoliberal policy conditions after 1990. Homelessness in Vancouver was unheard of in the early 1980s. At last count, in 2019 it had reached 2,223 people.^143^ This study of public health’s changing role in housing adds an intimate level of detail to the wider arc of the story of homelessness. While Rental-RRAP had been restored by the federal government under the Liberals in 1994, it was never funded to the same extent, nor coordinated to the degree that had been achieved by DHUP in the late 1980s.^144^ By the mid-1990s, the brief success of a new stick and carrot paradigm of the Rental-RRAP program in the DTES was long forgotten and stand-alone municipal efforts to enforce its bylaws have failed to prevent SRO decline.

The final nail in the coffin of public health policy abandonment in Vancouver occurred soon after. Following the lead of the neighbouring province of Alberta, BC undertook a decade-long process of neoliberal health care restructuring starting in 1993. The 2001 creation of a regional health authority, Vancouver Coastal Health (VCH), stripped municipal oversight over public health altogether. A critical perspective demonstrates how this restructuring was designed to place provincial ministries at a political distance from the burden of escalating health care costs, wait times, and overcrowded hospitals.^145^ In the context of the DTES, regionalization has had the effect of obfuscating who, between the city and the province, should take responsibility for the public health disaster that has unfolded in the neighborhood. The removal of a public health presence from the municipal apparatus has created a permanent governmental schism between institutional public health leadership vested in the province and the municipal oversight of the built environment.

For its part, VCH, reflecting a public health paradigm focused on population-based data and the individualization of risks, has largely focused its attention in the DTES on substance use and communicable disease prevention, followed perhaps by the recognition of the mental health crisis within the DTES population. For years, housing habitability has been left largely to municipal inspectors with no public health qualifications, who have been either unwilling or incapable of responding to the full scale of the health crisis happening in and around SRO buildings. VCH inspectors respond to housing complaints only when called upon by city inspectors. Their presence in DTES SRO housing inspection has been ad hoc and largely ineffectual.

The abandonment of the historical role of public health in the colonial governance of sanitary health in favor of an equally colonial ethos of epidemiological evidence and behavioral persuasion consistent with the “new public health” expresses the neoliberal episteme. Meanwhile, both within and beyond the SROs, the DTES is ground zero of the current opioid epidemic, with mortality rates compounded by the adverse living conditions in the neighborhood. It is questionable whether the conditions of life for the 4,500 people currently living in the remaining 104 privately owned SROs, one-third of whom self-report as Indigenous, are “better” than that of the 2,500 occupying shelters and living on the streets.^146^ The extent of adversity has been brought sharply into focus with the advent of the COVID-19 crisis, which has amplified already untenable housing conditions. The Right to Remain Research Collective within which this study has been embedded represents one attempt to help public health to “unlearn” its recent history, and hopefully to “relearn” the importance of protecting the housing rights of SRO tenants in Vancouver as a bona fide determinant of health.

## Notes

1. “Slave Labor,” *The Vancouver Sun*, March 16, 1987, p. B4; Larry Pynn, “On Seedy Side of the Street, It’s Well, Seedy: BALMORAL Life on Vancouver’s Skid Road Remains the Same: A Scummy and Nasty Business,” *The Vancouver Sun*, October 3, 1992, p. A1; Lori Culbert, “Hotels Charge More Than Welfare Allows,” *The Vancouver Sun*, June 19, 2009, p. A10.

2. Mike Hager, “Vancouver Seeks Further Charges against Regent Hotel Owners,” *The Globe and Mail*, December 12, 2017, https://www.theglobeandmail.com/news/british-columbia/vancouver-seeks-further-charges-against-hotel-owners/article37311022/; Wendy Stueck and Mike Hager, “For Low-Income Residents in Vancouver, a Different Kind of Real Estate Crisis,” *The Globe and Mail*, May 28, 2018, https://www.theglobeandmail.com/canada/british-columbia/article-for-low-income-residents-in-vancouver-a-different-kind-of-real-estate/; Wendy Stueck and Mike Hager, “Vancouver Orders Closing of Sahota-Owned Regent Hotel, Plans Purchase,” *The Globe and Mail*, June 20, 2018, https://www.theglobeandmail.com/canada/british-columbia/article-vancouver-orders-closing-of-sahota-owned-regent-hotel-plans-purchase/.

3. Stueck and Hager, “Vancouver Orders.”

4. Caitlin Shane and Pivot Legal Society, *Re: Section 23.11 of the Standards of Maintenance Bylaw*, Legal briefing, August 31, 2017.

5. The Downtown Eastside SRO Collaborative Society, The Lowdown on Upkeep: A Report on Tenants’ Experiences Living in Vancouver’s SROs, Report, November 2019.

6. Allan McConnell, “What Is Policy Failure? A Primer to Help Navigate the Maze,” *Public Policy and Administration* 30, no. 3-4 (2015): 221-42, https://journals.sagepub.com/doi/10.1177/0952076714565416; Mark Bovens and Paul ‘t Hart, “Revisiting the Study of Policy Failures,” *Journal of European Public Policy* 23, no. 5 (2016): 1350-763, https://www.tandfonline.com/doi/full/10.1080/13501763.2015.1127273.

7. Daniel J. Wilson, “The Limits of Public Health Reform in Urban America,” *Journal of Urban History* 42, no. 4 (2016): 800-805, https://journals.sagepub.com/doi/abs/10.1177/0096144216645785?journalCode=juha.

8. See Nick Blomley & the Right to Remain Collective. Producing the outlaw zone: property law and the geographies of relegation. Forthcoming in the *International Journal of Urban and Regional Research*.

9. Alison Bashford, *Imperial Hygiene: A Critical History of Colonialism, Nationalism and Public Health* (Houndsmills and New York: Palgrave Macmillan, 2004).

10. Ibid.

11. Nikolas Rose and Peter Miller, “Political Power beyond the State: Problematics of Government,” *The British Journal of Sociology* 43, no. 2 (1992): 173-205.

12. Skid Road (v. Row) is the historically accurate toponym in Vancouver as in other cities of the Pacific Northwest.

13. Barry Doyle, “Ideology, Discourse, and Locality: New Histories of Urban Public Health,” *Journal of Urban History* 31, no. 2 (January 1, 2005): 241-48, https://journals.sagepub.com/doi/abs/10.1177/0096144204265205?journalCode=juha.

14. Dorothy Porter, *The History of Public Health and the Modern State* (Amsterdam: Editions Rodopi, 1994).

15. Bashford, *Imperial Hygiene*.

16. Jill Wade, “Home or Homelessness? Marginal Housing in Vancouver, 1886–1950,” *Urban History Review/Revue d’histoire urbaine* 25, no. 2 (1997): 19-29, https://www.jstor.org/stable/43552094?seq=1#metadata_info_tab_contents.

17. Bashford, *Imperial Hygiene*.

18. Susan Craddock, “Embodying Place: Pathologizing Chinese and Chinatown in Nineteenth-Century San Francisco,” *Antipode* 31, no. 4 (October 1999): 351-71, https://onlinelibrary.wiley.com/doi/full/10.1111/1467-8330.00109; Bashford, *Imperial Hygiene*.

19. Wade, “Home or Homelessness?”

20. Patricia E. Roy, *A White Man’s Province: British Columbia Politicians and Chinese and Japanese Immigrants, 1858-1914* (University of British Columbia Press, 1990), 13.

21. Kay Anderson, “The Idea of Chinatown: The Power of Place and Institutional Practice in the Making of a Racial Category,” *Annals of the Association of American Geographers* 77, no. 4 (1987): 580-98, https://www.tandfonline.com/doi/abs/10.1111/j.1467-8306.1987.tb00182.x.

22. Roy, *White Man’s Province*.

23. Margaret W. Andrews, “The Emergence of Bureaucracy: The Vancouver Health Department,” *Journal of Urban History* 12, no. 2 (1986): 131-46, https://journals.sagepub.com/doi/abs/10.1177/009614428601200203.

24. Andrews, “The Emergence of Bureaucracy.”

25. Vancouver Environmental Health Division, Archival File Re Lodging Houses, 1913-1927, COV-S69, Box 145-C-01, Folder 01, City of Vancouver fonds, City of Vancouver Archives, Vancouver, British Columbia, Canada (hereafter referred to as COVA).

26. Andrews, “The Emergence of Bureaucracy.”

27. Vancouver Environmental Health Division, Archival file re: Lodging houses, 1913-1927, COVA.

28. Andrews, “The Emergence of Bureaucracy.”

29. Patricia E. Roy, *The Oriental Question: Consolidating a White Man’s Province, 1914-41* (Vancouver: University of British Columbia Press, 2011).

30. Vancouver License and Business Tax Office, General License Register: Lodging Houses, 1939, COV-S-383, Box 126-C-02, Folder 01-03. City of Vancouver fonds, COVA; Very few Chinese names are listed in the city’s Registry of Lodging House Business Licenses in 1938-1939. Jill Wade notes that a majority of Lodging Houses were unlicensed, suggesting this was more the case among Chinese managed businesses.

31. Shlomo Hasson and David Ley, *Neighbourhood Organizations and the Welfare State* (Toronto: University of Toronto Press, 1994); Anderson, “The Idea of Chinatown.”

32. Vancouver Office of the City Clerk, Japanese Property—Powell Street, 1942, COV-S27, Box: 027-E-06, Folder 17, City of Vancouver fonds, COVA.

33. Vancouver License and Business Tax Office, General License Register: Lodging Houses, 1939, COVA.

34. “Whites Already Taking Over Evacuated Homes in Japtown,” *The Vancouver Sun*, July 15, 1942, p. 15.

35. By 1950, conditions in the core area had deteriorated to the point that the Lodging Bylaw was amended to specifically include hotels and cabins, as many of the historic hotels in the area were by then largely accommodating permanent residents. “New City Lodging Bylaw,” *Vancouver Daily Province*, April 22, 1950, p. 2.

36. Larry Campbell, Neil Boyd, and Lori Culbert, *A Thousand Dreams: Vancouver’s Downtown Eastside and the Fight for Its Future* (Vancouver: Greystone Books, 2009), 12.

37. Vancouver Community Development Department, Downtown East Side, 1971, PUB-: PD 899, Box 148-A-02, City of Vancouver fonds, COVA.

38. Stewart Murray, “Administration of the Greater Vancouver Metropolitan Health Services,” *Canadian Journal of Public Health* 43, no. 5 (May 1, 1952): 202-208, https://www.jstor.org/stable/41980325?seq=1#metadata_info_tab_contents; Megan J. Davies, “Competent Professionals and Modern Methods: State Medicine in British Columbia during the 1930s,” *Bulletin of the History of Medicine* 76, no. 1 (April 1, 2002): 56-83, https://muse.jhu.edu/article/4820.

39. Murray, “Administration of the Greater.”

40. Vancouver Environmental Health Division, Subseries 2—Correspondence, Housing, 1930-1966, COV-S71-2. City of Vancouver fonds, COVA.

41. Office of the City Clerk, Report to Council of the Standing Committee of Council on Community Services, B.C. March 22/April 3, 1979, COV-S31-F131.15, Box: 897-E-03, Folder 06. City of Vancouver fonds, COVA.

42. J. A. Stringer, Sanitation Section Priority Lists, September 27, 1973, COV-S71-6, Box: 129-C-02, Folder 05, City of Vancouver fonds, COVA.

43. Vancouver Environmental Health Division, Inspector’s Reports, 1967-1976, COV-S69, Box: 145-B-07, Folder 06. City of Vancouver fonds, COVA.

44. Ibid.

45. Hasson and Ley, *Neighbourhood Organizations*.

46. Reva Dexter, Equal Opportunity Program Departmental Status Profile, July 20,1982, COV-S643, Box 138-F-3, Folder 1-4, City of Vancouver fonds, COVA.

47. Vancouver Planning Department and Vancouver Town Planning Commission, “Urban Renewal—Social Planning [i.e. Social Aspects],” 1965-1970, COV-S648, Box: 926-E-01, Folder 02, City of Vancouver fonds, COVA.

48. Ibid.

49. “Suburban News,” *The Vancouver Sun*, June 23, 1967.

50. C. A. Gill, A Report on Skid Road, August 1969, PUB-: PD 275, Box 147-C-02, City of Vancouver fonds, COVA, p. 3.

51. Vancouver Planning Department and Vancouver Town Planning Commission, “Urban Renewal—Social Planning [i.e. Social Aspects],” 1965-1970, COVA.

52. Office of the City Clerk, Information submitted by the Vancouver Board of Administration to the Standing Committee on Planning and Development, May 7, 1970, COV-S20, Box 018-G-05, Folder 07, City of Vancouver fonds, COVA.

53. Ibid.

54. Vancouver Community Development Department, Downtown East Side, 1971, COVA.

55. Ibid.

56. Vancouver Community Development Department, Downtown East Side, 1971, COVA.

57. Ibid.

58. Gary Bannerman, “Poor Housing Facing Trial,” *The Province*, November 26, 1971.

59. Ibid.

60. Ibid.

61. “Crackdown Shuts 15 Flophouses,” *The Province*, February 10, 1972, p. 31.

62. G. H. Bonham, Letter from G. H Bonham to A. S. McMorran and Schedule “A,” December 7, 1973, City of Vancouver fonds, COV-S69, Box 145-C-01, Folder 11. City of Vancouver fonds, COVA.

63. Ibid.

64. Vancouver Environmental Health Division, File—Downtown Eastside Housing, 1970-1973, COVA.

65. Vancouver Community Development Department, Downtown East Side, 1971, COVA.

66. Hasson and Ley, *Neighbourhood Organizations*.

67. Vancouver Environmental Health Division, File—Downtown Eastside Housing, 1970-1973, COVA.

68. Ibid.

69. Ibid.

70. Ibid.

71. Ibid.

72. Vancouver Environmental Health Division, File—United Housing Fund, 1973, COV-S69, Box 145-C-02, Folder 16. City of Vancouver fonds, COVA.

73. Ibid.

74. City of Vancouver Bylaw No. 4738, *Lodging House Bylaw* (1973).

75. Hasson and Ley, *Neighbourhood Organizations*.

76. Personal Communication with Libby Davies, 2020.

77. Vancouver Health Department, File—Housing—Lodging House By-law enforcement, 1977-1979, COV-S132-4, Box 129-G-02, Folder 02, City of Vancouver fonds, COVA.

78. Vancouver Health Department, File—Housing—Lodging House By-law enforcement, 1977-1979, COVA.

79. Vancouver Health Department, File—Housing—Lodging House By-law enforcement, 1977-1979, COVA.

80. Harry Rankin, “Law Protects Slumlord’s Property, Not People,” *The Downtown East*, November, 1975, p. 7.

81. Personal Communication with Libby Davies, 2020.

82. Enforcement Rankin, “Law Protects Slumlord’s Property, Not People.”

83. Ibid.

84. Vancouver Health Department, File—Housing—Lodging House By-law enforcement, 1977-1979, COVA.

85. Ibid.

86. Dale Falkenhagen and Canada Mortgage and Housing Corporation, *The History of Canada’s Residential Rehabilitation Assistance Program (RRAP)/Prepared by Dale Falkenhagen for Canada Mortgage and Housing Corporation* (Ottawa: Canada Mortgage and Housing Corporation, 2001); Hasson and Ley, *Neighbourhood Organizations*.

87. Bruce Eriksen, Letter from Bruce Eriksen to Barney Danson, May 6 1976, R13905-0-X-E, Vol 22, File 50, Library and Archives Canada, Barney Danson fonds, Ottawa, Ontario, Canada; Residential funding—Downtown Eastside, 1975-1978, COV S62-8, Box 10-F-5, Folder 3, City of Vancouver fonds, City of Vancouver Archives, Vancouver, British Columbia, Canada; “Danson Waffles on Slum Upgrading,” *The Downtown East*, July 1976.

88. Vancouver Health Department, Downtown Eastside Residents’ Association, 1977-1979, COV-S643, Box 087-B-05, Folder 08, City of Vancouver fonds, COVA.

89. “DERA Wants Inspectors,” *The Downtown East*, July 1977.

90. Vancouver Health Department, File—Housing—Lodging House By-law enforcement, 1977-1979, COVA.

91. Ibid.

92. “DERA Wants Inspectors.”

93. “City Council Okays 3 More Health Inspectors,” *The Downtown East*, August 1977.

94. Vancouver City Manager, Report to City Council, March 7, 1978, COV-S31-F128.12, Box 897-D-07, Folder 10, City of Vancouver fonds, COVA; Randy Glover, “Slum Owners Get Breather as Council Dithers,” *The Vancouver Sun*, March 18, 1978.

95. “DERA Wants Inspectors”; Glover, “Slum Owners.”

96. Vancouver Health Department, File—Housing—Lodging House By-law enforcement, 1977-1979, COVA.

97. Vancouver Office of the City Clerk, Council Meeting Minutes, November 7, 1978, COV-S31-F130.12, Box: 897-E-02, Folder 06, City of Vancouver fonds, COVA.

98. Office of the City Clerk, Report to Council of the Standing Committee of Council on Community Services, March 22/April 3, 1979, COV-S31-F131.15, Box: 897-E-03 Folder 06, City of Vancouver fonds, COVA.

99. Office of the City Clerk, City Council Meeting Minutes, November 7, 1978, COVA.

100. Ibid.

101. Vancouver Office of the City Clerk, Policy Advisory Committee Minutes, November 9,1978, COV-S643, Box 87-C-5, Folder 12. City of Vancouver fonds, COVA.

102. Vancouver Office of the City Clerk, Policy Advisory Committee Minutes, February 22, 1979, COV-S643, Box 94-B-1, Folder 3, City of Vancouver fonds, COVA.

103. Vancouver Office of the City Clerk, Report to Council of the Standing Committee of Council on Community Services, 1979, COVA.

104. Vancouver Social Planning Department, Downtown Housing Implementation Committee, 1979-1983, COV-S571, Box 101-D-01, Folder 01, City of Vancouver fonds, COVA.

105. Vancouver Health Department, File—Housing—Lodging House By-law enforcement, 1977-1979, COVA.

106. Ibid., p. 80.

107. Ibid.

108. Vancouver Health Department, File—Housing—Lodging House By-law enforcement, 1977-1979, COVA.

109. Ibid.

110. Ibid.; City Clerk, Extract from the Minutes of the Vancouver City Council Meeting, August 12, 1980, COV-S571, Box: 101-D-01, Folder 01, City of Vancouver fonds, COVA.

111. Ibid.

112. Tim Roark, Letter to Mayor Volrich and Members of Council, April 9, 1979, COV-S643, Box 243-B-05, Folder 06, City of Vancouver fonds, COVA, p. 1.

113. Vancouver Social Planning Department, Downtown Housing Implementation Committee, 1979-1983, COV-S571, Box 101-D-01, Folder 01, City of Vancouver fonds, COVA.

114. Vancouver Social Planning Department, Downtown Housing Implementation Committee, 1979-1983, COVA.

115. Ibid.

116. Vancouver Office of the City Clerk, City Council Meeting Minutes, July 8, 1978, COV-S31-F120.12, Box 897-C-07, Folder 2, City of Vancouver fonds, COVA.

117. Vancouver Social Planning Department, Downtown Housing Implementation Committee, 1979-1983, COVA.

118. Vancouver Office of the City Clerk, File—Residential funding—Residential Rehabilitation Assistance Program (RRAP), 1988-1990, COV S62, Box 937-B-1, Folder 3, City of Vancouver fonds, COVA; Jim Green, Stephen Learey, Sue Harris, Anna Wong, and Moon Yee, *Downtown Eastside Housing and Residents Survey, 1987-1988* (Vancouver and Ottawa: Canada Mortgage and Housing Corporation, 1989).

119. Ibid.

120. Parliament of Canada, Budget Papers Tabled in the House of Commons by the Honorable Michael H. Wilson, Minister of Finance, Ottawa, Government of Canada, April 27, 1989.

121. Vancouver Office of the City Clerk, File—Residential funding—Residential Rehabilitation Assistance Program (RRAP), 1988-1990, COVA.

122. Ibid.

123. Ibid.

124. Ibid.

125. Ibid.

126. Ariella Azoulay, *Potential History: Unlearning Imperialism* (Brooklyn: Verso, 2019).

127. Bashford, *Imperial Hygiene*, 11.

128. Ann Robertson, “Shifting Discourses on Health in Canada: From Health Promotion to Population Health,” *Health Promotion International* 13, no. 2 (January 1, 1998): 155-66, https://academic.oup.com/heapro/article/13/2/155/615396.

129. Marc Lalonde, *A New Perspective on the Health of Canadians: A Working Document* (Ottawa: Information Canada, 1975).

130. Barbara Legowski and Lindsey McKay, *Health beyond Health Care: Twenty-Five Years of Federal Health Policy Development* (Ottawa: Canadian Policy Research Networks, 2000).

131. Ibid.

132. Vancouver Office of the City Clerk, City of Vancouver Review of Department Activities and Budgets, 1977: Health Department, City of Vancouver, November 3, 1977, COV-S40, Box 120-G-01, Folder 90, City of Vancouver fonds, COVA.

133. Ibid., p. 8.

134. Vancouver MHO, You, Vancouver: What Your City Health Department Knows and Does, 1976, PUB-: PD 914, Box: 148-A-02, City Publications Collection, COVA, p. 1.

135. Vancouver Health Department, Vancouver, Health: 1980, 1980, COV-S643, Box 243-B-05, Folder 08, City of Vancouver fonds, COVA.

136. Jan O’Brien, “‘Visionary’ Lost Sight of Realpolitik,” *The Province*, February 6, 1983, p. B2.

137. Vancouver Health Department. “Vancouver, Health: 1980,” 1980, COVA.

138. Fritz Bowers. Memo from the City Manager to Health Department Employees, December 13, 1982, COV-S643, Box 243-A-2, Folder 2, City of Vancouver fonds, COVA.

139. Fran Cluett, Memorandum to Sharon Clark, July 20,1983, COV-S643, Box 138-F-3, Folder 14, City of Vancouver fonds, COVA.

140. H. E. McLean, “Memo to Management Committee Members, Professional Seniors and Program Coordinators,” August 11, 1983, COV-S643, Box 243-B-03, Folder 08, City of Vancouver fonds, COVA.

141. Personal communication with Dr. John Blatherwick, 2019.

142. Vancouver Social Planning Department, Downtown Housing Implementation Committee, 1979-1983, COVA.

143. The Homeless Services Association of B.C., The B.C. Non-Profit Housing Association, and CCC Urban Matters, *Vancouver Homeless Count 2019*, Vancouver, B.C., 2019.

144. Greg Suttor, *Still Renovating: A History of Canadian Social Housing Policy* (Montreal: McGill-Queen’s University Press, 2016).

145. Jonathan Lomas, John Woods, and Gerry Veenstra, “Devolving Authority for Health Care in Canada’s Provinces: 1. An Introduction to the Issues,” *Canadian Medical Association Journal* 156, no. 3 (February 1, 1997): 371-77, https://www.cmaj.ca/content/156/3/371; Jeffrey R. Masuda, Kerry Robinson, Susan J. Elliott, and John Eyles, “Chronic Disease Prevention and the Politics of Scale: Lessons from Canadian Health Reform,” *Social Work in Public Health* 27, no. 7 (November 1, 2012): 639-57, https://www.tandfonline.com/doi/abs/10.1080/19371910903182880; David Clark, “Neoliberalism and Public Service Reform: Canada in Comparative Perspective,” *Canadian Journal of Political Science* 35, no. 4 (December 2002): 771-93, https://www.jstor.org/stable/3233289?seq=1#metadata_info_tab_contents.

146. The DTES SRO Collaborative, *The Lowdown on Upkeep*.

